# Recommendations for laboratory workflow that better support centralised amalgamation of genomic variant data: findings from CanVIG-UK national molecular laboratory survey

**DOI:** 10.1136/jmg-2023-109645

**Published:** 2023-12-22

**Authors:** Sophie Allen, Lucy Loong, Alice Garrett, Bethany Torr, Miranda Durkie, James Drummond, Alison Callaway, Rachel Robinson, George J Burghel, Helen Hanson, Joanne Field, Trudi McDevitt, Terri P McVeigh, Tina Bedenham, Christopher Bowles, Kirsty Bradshaw, Claire Brooks, Samantha Butler, Juan Carlos Del Rey Jimenez, Lorraine Hawkes, Victoria Stinton, Suzanne MacMahon, Martina Owens, Sheila Palmer-Smith, Kenneth Smith, James Tellez, Mikel Valganon-Petrizan, Erik Waskiewicz, Michael Yau, Diana M Eccles, Marc Tischkowitz, Shilpi Goel, Fiona McRonald, Antonis C Antoniou, Eva Morris, Steven Hardy, Clare Turnbull

**Affiliations:** 1 Division of Genetics and Epidemiology, Institute of Cancer Research, Sutton, UK; 2 Department of Clinical Genetics, St George's University Hospitals NHS Foundation Trust, London, UK; 3 Sheffield Diagnostic Genetics Service, NEY Genomic Laboratory Hub, Sheffield Children's NHS Foundation Trust, Sheffield, UK; 4 East Anglian Medical Genetics Service, Cambridge University Hospitals NHS Foundation Trust, Cambridge, UK; 5 Wessex Regional Genetics Laboratory, University Hospital Southampton NHS Foundation Trust, Southampton, UK; 6 Yorkshire Regional Genetics Service, Leeds Teaching Hospitals NHS Trust, Leeds, UK; 7 Manchester Centre for Genomic Medicine and NW Laboratory Genetics Hub, Manchester University Hospitals NHS Foundation Trust, Manchester, UK; 8 Genomics and Molecular Medicine Service, Nottingham University Hospitals NHS Trust, Nottingham, UK; 9 Department of Clinical Genetics, CHI at Crumlin, Dublin, Ireland; 10 Cancer Genetics Unit, The Royal Marsden NHS Foundation Trust, London, UK; 11 West Midlands, Oxford and Wessex Genomic Laboratory Hub, Oxford University Hospitals NHS Foundation Trust, Oxford, UK; 12 Department of Molecular Genetics, Royal Devon and Exeter NHS Foundation Trust, Exeter, UK; 13 East Midlands and East of England Genomics Laboratory, Nottingham University Hospitals NHS Trust, Nottingham, UK; 14 North Thames Genomic Laboratory Hub, Great Ormond Street Hospital for Children NHS Foundation Trust, London, UK; 15 Central and South Genomic Laboratory Hub, Birmingham Women's and Children's NHS Foundation Trust, Birmingham, UK; 16 South East Genomics Laboratory Hub, Guy's Hospital, London, UK; 17 North West Genomic Laboratory Hub, Manchester Centre for Genomic Medicine, Manchester, UK; 18 Centre for Molecular Pathology, Institute of Cancer Research Sutton, Sutton, UK; 19 Department of Molecular Diagnostics, The Royal Marsden NHS Foundation Trust, London, UK; 20 Institute of Medical Genetics, Cardiff and Vale University Health Board, University Hospital of Wales, Cardiff, UK; 21 South West Genomic Laboratory Hub, University Hospitals Bristol NHS Foundation Trust, Bristol, UK; 22 North East and Yorkshire Genomic Laboratory Hub, Newcastle upon Tyne Hospitals NHS Foundation Trust, Newcastle upon Tyne, UK; 23 Cancer Sciences, Faculty of Medicine, University of Southampton, Southampton, UK; 24 Wessex Clinical Genetics Service, Princess Anne Hospital, Southampton, UK; 25 Department of Medical Genetics, National Institute for Health Research Cambridge Biomedical Research Centre, University of Cambridge, Cambridge, UK; 26 NHS England, National Disease Registration Service, London, UK; 27 Health Data Insight CIC, Cambridge, UK; 28 Department of Public Health and Primary Care, University of Cambridge Centre for Cancer Genetic Epidemiology, Cambridge, UK; 29 Nuffield Department of Population Health, University of Oxford, Oxford, UK

**Keywords:** Genetics, Genomics, Molecular Diagnostic Techniques, Clinical Laboratory Techniques, Genetic Testing

## Abstract

**Background:**

National and international amalgamation of genomic data offers opportunity for research and audit, including analyses enabling improved classification of variants of uncertain significance. Review of individual-level data from National Health Service (NHS) testing of cancer susceptibility genes (2002–2023) submitted to the National Disease Registration Service revealed heterogeneity across participating laboratories regarding (1) the structure, quality and completeness of submitted data, and (2) the ease with which that data could be assembled locally for submission.

**Methods:**

In May 2023, we undertook a closed online survey of 51 clinical scientists who provided consensus responses representing all 17 of 17 NHS molecular genetic laboratories in England and Wales which undertake NHS diagnostic analyses of cancer susceptibility genes. The survey included 18 questions relating to ‘next-generation sequencing workflow’ (11), ‘variant classification’ (3) and ‘phenotypical context’ (4).

**Results:**

Widely differing processes were reported for transfer of variant data into their local LIMS (Laboratory Information Management System), for the formatting in which the variants are stored in the LIMS and which classes of variants are retained in the local LIMS. Differing local provisions and workflow for variant classifications were also reported, including the resources provided and the mechanisms by which classifications are stored.

**Conclusion:**

The survey responses illustrate heterogeneous laboratory workflow for preparation of genomic variant data from local LIMS for centralised submission. Workflow is often labour-intensive and inefficient, involving multiple manual steps which introduce opportunities for error. These survey findings and adoption of the concomitant recommendations may support improvement in laboratory dataflows, better facilitating submission of data for central amalgamation.

**WHAT IS ALREADY KNOWN ON THIS TOPIC**

- The principles of genomic laboratory workflow have been described at high level in previous publications.

**WHAT THIS STUDY ADDS**

- This first national survey of genomic data workflow conducted in 2023 reflects in detail practices in 17 National Health Service molecular genetics laboratories in England and Wales.

**HOW THIS STUDY MIGHT AFFECT RESEARCH, PRACTICE OR POLICY**

- The survey responses illustrate laboratory workflow for preparation of genomic variant data for centralised submission that is frequently labour-intensive, highly manual and inefficient. These findings may be instructive to laboratories for improving dataflows which better enable downstream submission of data for national amalgamation endeavours.

## Introduction

Over the last decade, there has been a substantial, national-level focus on expanding the role of genomics in routine National Health Service (NHS) care, with the goal of clinical services ‘operating to national standards, specifications and protocols’.^1–3^ To deliver this transformation, NHS England has reconfigured the 28 English molecular diagnostic laboratories into seven Genomic Laboratory Hubs (GLHs) with development of a National Test Directory for each clinical indication (denoted by an ‘R-code’) determining germline test eligibility criteria, gene panels and constituent molecular analyses for each genomic test.^4^

Underpinning this transformation, and key to expansion of genomic testing capacity, will be the data systems by which the genomic data are generated, processed, analysed and stored. Upstream workflow can be wholly automated, with high throughput conversion of image data from next-generation sequencing (NGS) into output variant call format (VCF) files listing the called genomic variants, with structured fields for the genomic location, variant nomenclature and quality metrics. This workflow has now been widely and successfully implemented by clinical bioinformaticians across NHS molecular diagnostic laboratories.

However, downstream of this are unavoidably more manual processes requiring expert evaluation of the detected germline variants by experienced clinical diagnostic scientists for (1) technical veracity and (2) pathogenicity classification. To confirm the variant is truly present, the sequence data may require manual inspection and further molecular analysis using an orthogonal technology to validate the called variant (for example, Multiplex Ligation-dependant Probe Amplification to confirm an exon-level deletion). While pre-established filters can be applied to remove many variants that are likely benign, manual evaluation of pathogenicity must be undertaken for the remaining variants identified. This requires assembly of information from multiple sources, including variant effect prediction, *in silico* protein function effect prediction, population frequencies (eg, gnomAD, UK Biobank), functional assay results, case–control study data, familial co-segregation data, previous classifications (eg, ClinVar, the Human Gene Mutation Database, local records) and review of the literature for phenotypical case descriptions.^5–10^ The assembled evidence must then be compared with the (continually evolving) generic and gene-specific protocols which dictate the scoring of evidence elements, along with rules defining combination of evidence scores, in order to produce a final classification.^11–15^ Only variants for which there is sufficient evidence for classification as ‘pathogenic’ (class 5) or ‘likely pathogenic’ (class 4) will be included in the diagnostic laboratory report for return to the clinician (and often patient). The majority of manually reviewed variants will not have attained sufficient evidence and these ‘variants of uncertain significance’ (VUSs, class 3) will not typically be included in the clinical report, unless the variant is on the threshold of uplift to likely pathogenic and meets the specific national criteria for being reported (as per the Association of Clinical Genomic Science (ACGS) Best Practice Guidelines).^16^ Because of the dynamic nature of the available evidence and associated guidance, after the elapse of a defined time period, new observations of previously evaluated variants require fresh review and (re)classification; national approaches have been agreed for national alerts and reissuing reports on clinically important reclassification of a variant.^17^

After variant classification and generation of the clinical report, the detected variants will typically then be transferred for long-term storage into the local Laboratory Information Management System (LIMS) and, variably, into the hospital-level Electronic Health Record (EHR) system. In an LIMS or EHR, the variant data are associated with patient identifiers (ie, NHS number, date of birth, name) and additional patient information (eg, test indication, ethnicity, age and sometimes phenotype). VCF files may also be stored but most often only the ‘SampleID’ and/or ‘RunID’ will be available within VCF files; typically there are no patient identifiers or patient information available in the VCF. The workflow through which variant data pass, and the stored data items and formats, are potentially critical to the readiness and fidelity by which these local data can be shared and amalgamated.

National amalgamation of genomic data is important to advance broader, global understanding of variant pathogenicity to reduce classifications of VUS. Assessing the frequency, familial segregation and phenotypical associations with which a variant is observed adds important evidence regarding the pathogenicity of a variant. Local amalgamations of accrued observations of a given variant may on occasion be informative, but it is typically only with national or international amalgamation of data on rare variants that we have sufficient instances by which to evaluate their association with clinical disease. Thus, along with ensuring robust local recording and reporting, LIMS/EHRs would ideally be designed to readily enable submission of local de-identified variant and phenotype data for national/international amalgamation.

In just such an endeavour, an informatic pipeline enabling local submission and national amalgamation of pseudonymised individual-level variant data for cancer susceptibility genes (CSGs) has been established by the NHS National Disease Registration Service (NDRS). Since 2018, all 16 English NHS laboratories undertaking CSG analyses have submitted locally held individual-level data on all germline CSG tests performed and variants detected. Data are then restructured and extracted at NDRS using bespoke laboratory-specific informatic processing algorithms developed by NDRS.^18^ This endeavour has, for the first time, allowed successful assembly of nationally complete individual-level data on genetic tests and detected variants, dating back for some laboratories as far as 2002. These data provide opportunities for variant interpretation and also longitudinal study of CSG variant carriers via linkage to cancer registrations. However, this activity has revealed considerable heterogeneity across participating laboratories regarding (1) the structure, quality and completeness of submitted data, and (2) the ease with which those data could be assembled locally for submission.

The Cancer Variant Interpretation Group UK (CanVIG-UK) was established in 2017, at the directive of ACGS, to coordinate interpretation of variants in CSGs.^15^ CanVIG-UK meets monthly and comprises >300 members, including clinical scientists and genetics clinicians from each of the seven English GLHs (in addition to those from the devolved nations and Ireland). CanVIG-UK has since inception worked in close partnership with NDRS to coordinate laboratory data submissions, and also in the analysis and dissemination of the NDRS nationally amalgamated variant data.

The combined bioinformatic and human workflow through which variant data in VCFs are evaluated, classified, transferred to local LIMS/EHRs and eventually stored has been demonstrated to be critical to the feasibility and utility of the NDRS national data amalgamation. To better understand this workflow, through CanVIG-UK, we conducted a survey of the 17 individual English and Welsh laboratories which perform molecular diagnostic germline testing in CSGs.

## Methods

The survey questions were designed and piloted by the CanVIG-UK Steering and Advisory Group, which comprises eight senior clinical scientists and three consultant clinical geneticists working in Cancer Genetics across the GLHs (online supplemental table 1). The closed online survey was then sent to up to three CanVIG-UK clinical scientists undertaking germline cancer susceptibility genetic analyses from each of the 16 laboratories in which diagnostic CSG testing in the GLH network is performed, as well as one laboratory in Wales. Return of a single consensus response representing each laboratory was requested in June 2023 (see online supplemental methods). Complete responses were returned by 17 of 17 laboratories surveyed.

10.1136/jmg-2023-109645.supp3 Supplementary data

10.1136/jmg-2023-109645.supp2 Supplementary data

The survey comprised 18 questions relating to ‘NGS workflow’ (11), ‘variant classification’ (3) and ‘phenotypical context’ (4) (summary details of all questions and responses from responding laboratories are presented in online supplemental table 2); there were a further five questions relating to ‘respondent details’ and a separate component comprising 12 questions relating to very specific logistical aspects of ‘GLH-NDRS centralised data submission’.^19^

This survey was designed to assess elements of the workflow relevant to NDRS data submission; we did not survey on the workflow used specifically for generation of the diagnostic clinical report.

## Results

### Variant workflow from the NGS outputs into the LIMS

There was substantial variation in the workflow for managing and documenting the process of technical verification and variant classification upstream of entering the variants onto the LIMS. The laboratories reported workflow by which one, two or sometimes three generations of intermediary VCF-derived files were generated and stored (online supplemental figure 1A). Variants listed on the VCF were typically associated with a sample ID (15 of 17) which was usually also available in the LIMS, but rarely with a patient name (3 of 17), and never with a Date of Birth (0 of 17) or NHS number (0 of 17) (online supplemental figure 1B).

10.1136/jmg-2023-109645.supp1 Supplementary data

**Figure 1:**
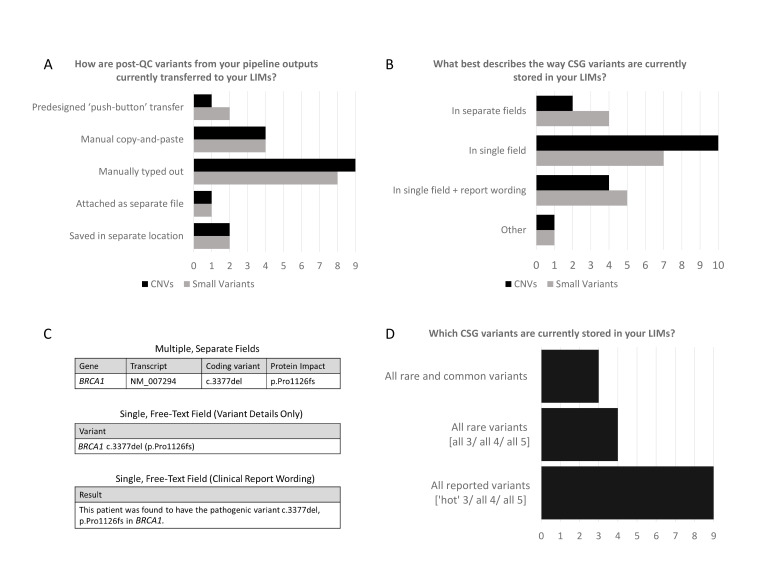
Responses from each laboratory for multiple choice questions pertaining to variant workflow from NGS pipelines to the LIMS. (A,B) All laboratory responses describing the method of transfer from NGS pipeline output to LIMS, and how this information is stored within the LIMS. (C) Visual description of differing LIMS storage formats. (D) All laboratory responses describing which detected variants are stored within an LIMS. CSG, cancer susceptibility gene; LIMS, Laboratory Information Management System; NGS, next-generation sequencing; QC, quality control.

Methods by which selected variants are entered into the LIMS largely rely on manual processes (figure 1A). Eight of 17 laboratories reported manually typing out the variant details, while 4 of 17 laboratories reported a manual ‘copy-and-paste’ mechanism for entering the variants into the LIMS. In two laboratories, the variant data could be entered into the LIMS via an automated ‘push-button' transfer from the NGS outputs (although only one of the systems serves both single nucleotide variants and CNVs). For one laboratory, the variant data were not entered in the LIMS but attached in the LIMS as a separate file. For two laboratories, the variant details were not stored in the LIMS at all but in a separate location outside of the LIMS.

Formatting of variant data within the LIMS was another key area by which laboratories differed (figure 1B). In 4 of 17 laboratories, small variant details were stored in the LIMS using separate structured fields for gene, cDNA and protein change (figure 1C). For 7 of 17 laboratories, these details were stored in a single field, while for 5 of 17 laboratories, the variant details were embedded within free text (typically comprising the full report wording). For CNVs, separate structured fields were even less frequently used (2 of 17) to store details of the variants.

Most laboratories decided which of the detected variants were selected for storage in the LIMS dependant on if the variant was reported clinically (figure 1D). Three of 17 laboratories reported that all variants (rare and common) were stored in their LIMS, while 4 of 17 laboratories reported that all rare variants were stored in their LIMS (regardless of pathogenicity class). However, for 9 of 17 laboratories, only variants included in their clinical report were stored in their LIMS, which will therefore only comprise variants classified as likely pathogenic/pathogenic and occasional ‘VUS’ of particularly high suspicion (so-called ‘hot’ VUS).^16^ Some of these laboratories reported parallel systems by which the other variants were stored against meaningful patient identifiers, for example, using an additional in-house Excel file or a separate database. When surveyed regarding their confidence that their local system would allow reliable variant retrieval in the event of a ‘cold’ VUS being upclassified into pathogenic, 13 were extremely or very confident, 3 were quite confident and 1 was not very confident (online supplemental figure 2).

### Variant classification

Also variable were the workflow and resources available to clinical scientists for variant classification, which is typically performed upstream of entry of the variant into the LIMS (table 1). In 8 of 17 laboratories, the variants requiring classification are viewed within a commercial or in-house ‘variant system’, while in 5 of 17 laboratories, the variants are viewed in a spreadsheet. Respondents from 9 of 17 laboratories reported that for the variants requiring classification, there would be minimal or no automatic annotations available in their workflow/system (beyond basic population frequencies), meaning that proactive manual accessing of multiple relevant data sources is required, such as Alamut, ClinVar and CanVar-UK. Respondents from 7 of 17 laboratories reported that their system provided variant-specific links out to most or many of the resources. Only one laboratory reported most/many of the relevant data resources being directly available within their variant system.

**Table 1:** Responses for questions surrounding variant classification and storage of such information; for these questions, respondents could select multiple options

**Interface for viewing variants requiring evaluation/classification**	**No of labs**
Within a bioinformatic processing system/dedicated in-house variant system	8
In a spreadsheet (eg, VCF, VCF-derived file)	5
Other	4
**Within the interface from which you view variants requiring interpretation, which description is most accurate?**	**No of labs**
Most/many of the relevant data sources have been pre-imported	1
There are variant-specific links out to most/many of the relevant data sources	7
No or minimal annotations (eg, only population frequencies). Accessing of relevant data sources (Alamut, CanVar-UK, ClinVar, literature) requires manual interrogation (variant name is typed/pasted in)	9
**Storage of variant evaluation/classification (laboratories may use more than one system**)	**No of labs**
Dedicated in-house departmental variant data system	5
Commercial platform or software (eg, Congenica, Alamut)	4
LIMS (against specific patient)	3
Individual per-variant files. File is updated on each encounter of the variant	5
Individual per-variant files. New file is generated each time the variant is encountered	4
Individual per-patient episode files. May contain multiple variants	4
Per-gene files comprising multiple variants	1
Per-disease files comprising multiple genes (and multiple variants)	1

LIMS, Laboratory Information Management System; VCF, variant call format.

Regarding storage of detailed variant classification findings, the primary divide was between laboratories storing classification details as individual files versus those incorporating this information within a database (table 1). Most common (5 of 17 laboratories) was some form of in-house variant database (separate from the LIMS). Four laboratories store this information in a commercial platform (eg, Alamut, Congenica systems). Otherwise, there was a mix of approaches including dynamic per-variant files (updated on each new observation of the variant) or per-variant-per-patient files (generating a new file for each observation of a variant). These variant files were then stored in various locations including local drives or as attachments to the LIMS. Some laboratories reported using multiple storage methods (online supplemental table 2).

### Phenotypical details and test context

**Figure 2:**
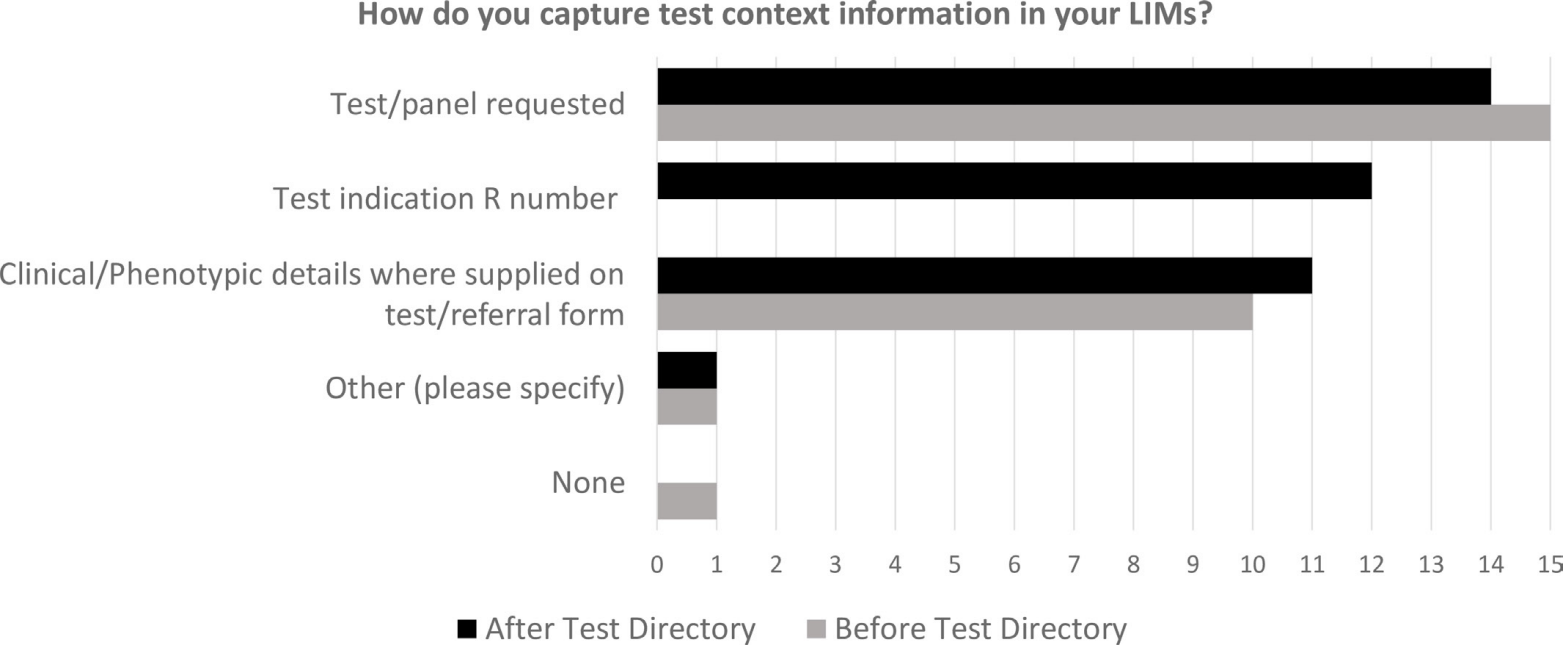
Comparison of testing context information captured by each laboratory before and after the National Test Directory was implemented. LIMS, Laboratory Information Management System.

In 15 of 17 laboratories, the details of the panel tested were specified in the LIMS (figure 2); this historically comprised series of gene names or local identifiers for their panels but since the introduction of the National Test Directory, 12 of 17 laboratories are routinely capturing the relevant clinical indication (R-code) in their LIMS. Only 11 of 17 laboratories record clinical details or phenotypical information in their LIMS (and this is only when information was provided on the request form). When small gene sets or single genes are reported from a larger panel (online supplemental table 2), a roughly equal number of laboratories reported listing the individual genes tested by name versus annotating with a subpanel name.

## Discussion

The responses from this survey describe the heterogeneous workflow used across the surveyed 17 laboratories for evaluating, classifying and storing germline variant data ahead of national centralised submission. Many of the reported elements in this workflow render potentially challenging and time-consuming the retrieval and centralised submission of variant data for national amalgamation. Workflow is often laborious and low-throughput, with manual steps which introduce opportunities for error. Given the commonality of many of these challenges across centres, we propose recommendations for workflow redesign that target the key challenges across workflow in variant data transfer, storage and retrieval.

### Variant workflow

The survey showed that the highly structured VCF file output from NGS is frequently processed downstream using non-automated approaches which generate multiple intermediary files, potentially introducing opportunity for human error with manual or copy–paste transcription of variants.

The existing laboratory processes were designed to ensure relevant results are included accurately on the clinical diagnostic report for each patient. They were not designed with large-scale national data amalgamation in mind. Consequently, the laboratories vary in whether they store rare variants (other than reported pathogenic and likely pathogenic variants) in their LIMS. Therefore, the majority of variants (especially rare VUS) may not universally be stored in the LIMS alongside patient identifiers and patient information. Instead, these variants might solely reside in stored VCF files which typically do not contain patient identifiers. Only with decryption using Sample and Run identifiers might these variant data be reunited with patient identifiers/phenotype information.

Our survey indicates that transformation of highly structured VCF variant data into the clinical information stored in an LIMS typically means that this final variant information (1) is for only a small subset of detected variants, (2) is in a variously less-structured format than a VCF (eg, free text) and (3) has potentially been subject to human transcription errors (although this has been shown to be infrequent).^18^ These three factors have the following implications: (1) they impact the ability to search for variants (when they are encountered by a laboratory again, or to identify patients with a certain variant), (2) the varied structures make data amalgamation challenging and (3) variable capture in local LIMS means the true frequency of rare VUSs in amalgamated data may be underestimated.

From the survey responses, the distilling of voluminous technically unverified variant data from a VCF across into clinically classified variants in an LIMS/EHR would appear to be a universal challenge, and few laboratories appear to have informatic systems within which this variant workflow can be comprehensively automated, executed and documented.

### Variant classification

The workflow reported for performing and capturing variant evaluation/classification was also highly heterogeneous and often labour-intensive. The reported workflow often necessitates not only accessing each of the required data sources manually on a variant-by-variant basis, but also then manual population of the findings in per-variant documents or files. Retrieval of per-variant files stored on local drives will also be subject to the inconsistencies of file-naming practices (especially where there is high staff turnover). Systems by which multiple per-patient-per-variant files for a given variant are maintained will also be potentially vulnerable to temporal inconsistencies.

### Phenotypical context and gene panels

The survey also revealed that there is substantial variation in the extent of capture of phenotype or testing context (panel name or constituent genes) against the detected variant(s) in the LIMS. The personal and/or familial phenotype and the context of the gene panel requested are highly informative regarding the likely clinical significance of a detected rare variant. For example, the new evidence towards pathogenicity is much greater for a rare *TP53* variant detected in a context with high phenotypical specificity (for example, testing of only one gene, *TP53*, in an adolescent with rhabdomyosarcoma and a family history of young-onset cancers) compared with a context with low phenotypical specificity (for example, testing of a 40-gene panel in a 68-year-old woman with breast cancer and no family history).^19 20^ ^21^ Without data on the tested gene panel and phenotype, the two instances of the variant would not be distinguishable. The value of variant data for longer-term advancement of risk estimation and variant classification locally, nationally or globally is greatly diminished if the concomitant phenotype data and testing context are not captured. Use of clinical indications (R-codes) from the National Test Directory will give some indication of testing context. However, the associated eligibility requirements for each R-code are broad and both these and the gene sets for a given code have changed considerably since inception of the National Test Directory in 2018 (and are likely to continue to change).

### Data sharing and national submission

There has been substantial global focus on improving national and international genomic data-sharing, with high-profile endeavours from the Global Alliance for Genomics and Health (GA4GH), such as the GA4GH ‘Beacon’ Project and the Matchmaker Exchange designed to enable cross-identification across the world of other instances of a given variant.^22–25^ In addition, the LOVD and ClinVar resources provide international portals by which clinical diagnostic laboratories can share observations and classifications of clinically observed variants.^7 26 27^ While submission to ClinVar is undertaken by CanVIG-UK for their consensus variant classifications, participation by individual laboratories in these international endeavours is potentially limited by the LIMS data architectures.^15^

The UK national CSG genomic data amalgamation is a world first—a reflection of the challenging logistics and the complexity of related governance structures. However, this has been achieved largely despite of rather than because of the design of the laboratory data systems and workflow. The utility of sharing of the variant data between laboratories is complemented by the national interlaboratory discussion forum afforded by the CanVar-UK platform, which provides opportunity for discussing how the variant frequencies are applied for variant interpretation, for sharing other de-identified clinical information (eg, tumour testing) and for discussing consensus variant classifications.

### Limitations of the survey

The laboratory workflow and practices are as reported from a single clinical scientist survey response for each laboratory. Although we contacted multiple clinical scientists per laboratory to facilitate consensus response, and we checked directly with respondents where there were any inconsistencies within the information supplied, there is opportunity for misinterpretation of questions or supply of erroneous responses. In addition, there is the possibility that questions or multiple choice options may be interpreted differently by different laboratories, or of difference in opinion between those working within the same laboratory. By piloting, we sought in our survey design to present for each question the most clear and relevant enumerations; nevertheless, additional information was provided as free text with many responses. This we have sought to share (where not identifiable).

## Recommendations

Workflow redesign in a laboratory can be a sizeable undertaking, especially if this involves tighter integration of upstream sequencer VCF workflow into the LIMS and/or redesign of LIMS data storage/outputting. From our survey findings, we identified the following as priority recommendations to be considered in any laboratory data workflow redesign:

1. Transfer of variant data across the workflow from the VCF to the LIMS/EHR should be automated (informatic). *This will reduce the risk of errors in variant names (introduced by manual and/or copy-and-paste transcription) and will ensure consistent variant nomenclature.*
2. Variants (including CNVs) should be stored in a structured format, with separate fields for genome build, transcript, gene, cDNA and p. protein annotation (to standardised Human Genome Variation Society (HGVS) recommendations^28^). *This will support both individual variant querying and broader amalgamation/analyses.*
3. All rare variants (preferably all variants) should be stored against the patient record, either within the primary LIMS or within a linked data system which contains meaningful patient identifiers. *This will ensure that relevant historical patients can be identified if a variant is reclassified or prospectively introduced for clinical testing.*
4. All rare variants should be stored against details of the clinical indication for testing/gene set analysed and (where possible) details of patient phenotype. *This will ensure that the variant data are most informative for variant interpretation.*
5. Local variant classifications (and the contributory evidence) should be stored as unique entries within a single structured data system (or within the LIMS) rather than as individual files. *This will improve data retrieval and reduce the occurrence of multiple discordant entries of the same variant. Where possible, and avoiding the release of patient-identifiable information, local variant classifications should be shared nationally (eg, CanVar-UK) and internationally (eg, ClinVar).*

## Conclusions

The reconfiguration of the NHS genomics laboratories, development of a National Test Directory, widening of testing indications and expansion of whole-genome sequencing offer potential for the UK to be a major contributor of national data to variant interpretation initiatives. However, this survey has revealed the workflow required for data amalgamation is frequently labour-intensive and potentially culminates in storage within the LIMS of variant data that may be incomplete, poorly structured, may incorporate rare manual transcription errors and lack corresponding phenotype data. Larger gene panels are being added to the National Test Directory and the volume of genetic testing in the NHS is increasing. With this volume increase, there is potential for current local and national data amalgamation processes to become compromised and for clinical diagnostic delivery to become increasingly burdensome, a particular concern given the current limited availability of trained clinical scientists.

The laboratory workflow taking VCFs into clinically processed variant data is inherently complex, with unavoidable requirement for human variant review. There is further complexity where integrated pathology reporting necessitates downstream integration of molecular genetics findings with results from other pathology disciplines (for example, in cancer reporting). Redesign of this workflow and LIMS architectures is therefore far from straightforward and will only be successful where consultative design involves substantial dedicated time from experienced clinical scientists and clinical bioinformaticians (who must be released from service delivery). However, considered investment in the redesign of this workflow will be of high value in empowering laboratory scientists for the proposed expansions in genomic analyses. It will also position the UK clinical genetic testing community to make the best use of the data generated to contribute to national and international initiatives in data amalgamation, thus supporting improved variation interpretation for our patients.

## Data Availability

Data are available in a public, open access repository. Results from the GLH-NDRS component of the full survey are available in the Zenodo repository at https://doi.org/10.5281/zenodo.8340397.
