# Supplementary material for: Recommendations for laboratory workflow that better support centralised amalgamation of genomic variant data: findings from CanVIG-UK national molecular laboratory survey
Source: J Med Genet. 2023 Dec 22;61(4):305–12. doi: 10.1136/jmg-2023-109645 (PMC10982625; doi:10.1136/jmg-2023-109645)
Supplement: Supplementary data [file jmg-2023-109645supp003.pdf]

Supplementary Table 1: Current CStAG Membership and representation across the UK GLHs

Genomic Laboratory Hub (GLH)	Number of CStAG Representatives
Central and South GLH	1
East GLH	2
North West GLH	1
North Thames GLH	2
South East GLH	2
South West GLH	0
North East and Yorkshire GLH	2
N/A - Ireland (Dublin)	1

Supplementary Table 2: Full response breakdown for the 18 questions pertaining to laboratory workflows. To preserve anonymity, free-text comments are not provided.

Question		Options	Number of Responses
1	Which of the following identifiers are present against a detected variant in the output of your NGS pipeline (VCF file and derived files)?	Hospital Trust ID	0
		NHS Number	0
		DOB	0
		Sample ID (not present in LIMs)	2
		Patient Name	3
		Patient Initials	3
		Run/NGS ID (not present in LIMs)	5
		Sex/Gender	5
		Run/NGS ID (that is also present in LIMs)	9
		Sample ID (that is also present in LIMs)	15
		Other	3
2	How are the QC'ed small variants (eg SNVs and indels) from your NGS pipeline outputs (VCF or VCF-derived file) CURRENTLY transferred to your LIMs?	Saved in separate location	2
		Attached as separate file	1
		Manual copy-and-paste	4
		Automatic and invisible (no action required from scientist)	0
		Manually typed out	8
		Predesigned 'push-button' transfer	2
		Other	0
3	For small variants: How many types/tiers of VCF-derived files do you generate?	>3	0
		3	2
		2	4
		1	10
		0	1
4	For small variants: How many types/tiers of VCF-derived files do you store?	>3	0
		3	1
		2	1
		1	13
		0	2
5	How are the QC'ed CNVs (eg exon-level deletion) from your pipeline outputs (from VCF, VCF-derived or MLPA workflow output) CURRENTLY transferred to your LIMs?	Saved in separate location	2
		Attached as separate file	1
		Manual copy-and-paste	4
		Automatic and invisible (no action required from scientist)	0
		Manually typed out	9
		Predesigned 'push-button' transfer	1
		Other	0
6	For CNVs: How many types/tiers of VCF-derived	>3	0

	files do you generate?	3	1
		2	0
		1	15
		0	1
7	For CNVs: How many types/tiers of VCF-derived files do you store?	>3	0
		3	1
		2	0
		1	14
		0	2
8	What best describes the way CSG small variants are CURRENTLY stored on your LIMs?	In a single free text/unstructured field (which also includes additional clinical report wording)	5
		In a single free text/unstructured field (just contains variant name)	5
		As a single field of strict formal hgvs notation, ie: gene + transcript + genomic location + coding (c.) change + protein (p.) change	2
		As separate fields for gene/transcript/genomic location/coding (c.) change/protein (p.) change	4
		Stored outside of a LIMs system	1
9	What best describes the way CSG CNVs are CURRENTLY stored on your LIMs?	In a single free text/unstructured field (which also includes additional clinical report wording)	4
		In a single free text/unstructured field (just contains variant name)	9
		As a single field of strict formal hgvs notation, ie: gene + transcript + genomic location + coding (c.) change + protein (p.) change	1
		As separate fields for gene/transcript/genomic location/coding (c.) change/protein (p.) change	2
		Stored outside of a LIMs system	1
10	Which CSG variants are CURRENTLY stored in your LIMs?	All variants detected (rare and common)	3
		All rare variants detected (all class 3 and above, may also include rare class 1/2)	2
		All rare variants detected (all class 3 and above, no rare class 1/2)	2
		Most rare variants of relevance (all 4/5 and most interesting VUS)	5
		Only variants included in the clinical report	4
		No variants stored in the LIMs / No LIMs system	1
11	In the event of a variant re-classification (for example, up-classification of a previous cold class 3), how confidently/readily could you identify all patients in whom that variant had been identified (since inception of your current system)? (if not using a LIMs system for this process, please specify which system would instead be used to identify all patients)	5: extremely: all variants are stored in LIMs/current storage system in accurate structured format	5
		4: very: all variants are stored in LIMs/current storage system but have been manually entered, so subject to typos	8
		3: quite: cold class 3 variant likely not in LIMs/current storage system. Would require a comprehensive search of various historic bioinformatics systems/VCFs/derived files but these are stored so as to be easily searchable	3
		2: not very: cold class 3 variant likely not in LIMs/current storage system. Would require a comprehensive search of various historic bioinformatics systems/VCFs/derived files which are stored in multiple locations	1
		1: poorly: cold class 3 variant likely not in LIMs/current storage system. Would require a comprehensive search of various historic bioinformatics systems/VCFs/derived files which are not readily accessible	0
12	From what type of interface are the variants requiring interpretation viewed?	Within a bioinformatics processing system/dedicated in-house variant system	8
		In a spreadsheet (eg VCF, VCF-derived file.)	5
		Other (please specify)	4
13	Within the interface from which you view variants requiring interpretation, which description is most accurate?	Most/many of the relevant data sources have been pre-imported	1
		There are variant-specific hyperlinks to most/many of the relevant data sources.	7
		No/minimal annotations (eg only population frequencies). Accessing of relevant data sources (Alamut, CanVar-UK, ClinVar, literature) requires manual interrogation (variant name is typed/pasted in).	9

		Other	0
14	Following interpretation of a CSG variant, where do you store your updated detailed findings/pathogenicity classification (eg scoring on ACMG sub-elements)?	Dedicated in-house departmental variant datasystem	5
		Individual per-VARIANT files (excel, word, other document). Updated for recurrent viewings of variant.	5
		Individual per-VARIANT files (excel, word, other document). New file time a variant is encountered.	4
		Individual per-PATIENT EPISODE files (excel, word, other document) (may contain multiple variants)	4
		Commercial platform or software (eg Congenica, Alamut)	4
		LIMs (against specific patient)	3
		Individual per-GENE files (excel, word, other document).	1
		Individual per-DISEASE files (excel, word, other document).	1
		LIMs (against variant)	0
		Dedicated single departmental excel/spreadsheet	0
		Variant classifications are only documented on the patient report and not elsewhere stored	0
		Other (please specify)	0
15	How did you capture test context information in your laboratory patient-level datastorage system (LIMs) prior to the GMS test directory?	Clinical details/Phenotype information from test request or referral form	10
		Test/panel requested	15
		None	1
		Other (please specify)	1
16	How do you capture test context information in your laboratory patient-level datastorage system (LIMs) since the GMS test directory implemented?	Clinical details/Phenotype information from test request form or referral form	11
		Test/panel requested	14
		Test indication R number (since publication of the National Genomic Test Directory)	12
		None	0
		Other (please specify)	1
17	Following implementation of the GMS test directory, please estimate what % of CSG test requests now contain some clinical details/phenotype information (in addition to test requested/R number).	0-24%	0
		25-49%	2
		50-74%	8
		75-100%	6
18	When a single gene/small gene set is reported from a larger panel/exome, how is the gene set which is analysed captured in your LIMs?	Genes analysed are listed by name in LIMs against patient (free text entry eg list with commas)	4
		Name of subpanel(s) listed in LIMs against patient (structured entry)	4
		Genes analysed are listed by name in LIMs against patient (structured entry eg selected from a drop-down list, imported from a separate portal)	2
		Name of subpanel(s) listed in LIMs against patient (free text entry)	2
		Mixture of genes and subpanels (free text entry)	2
		Genes/panels analysed not documented in LIMs; requires reference to other files within NGS workflow	1
		Exported as a list from commercial platform and attached to LIMs record	1
		Other (please specify)	1
