# Supplementary material for: Recommendations for laboratory workflow that better support centralised amalgamation of genomic variant data: findings from CanVIG-UK national molecular laboratory survey
Source: J Med Genet. 2023 Dec 22;61(4):305–12. doi: 10.1136/jmg-2023-109645 (PMC10982625; doi:10.1136/jmg-2023-109645)
Supplement: Supplementary data [file jmg-2023-109645supp002.pdf]

SUPPLEMENTARY METHODS

Initial creation of survey (Pilot Phase 1)

The initial survey questions and format were iterated and reviewed through discussion with the CanVIG-UK Steering and Advisory Group (CStAG), an expert panel supporting Cancer Variant Interpretation Group UK (CanVIG-UK) activities, and consisting of 8 senior clinical scientists and 3 consultant clinical geneticists from across the Genomic Laboratory Hubs.

After initial discussions, the survey was piloted by 4 CStAG members to assess the survey clarity and content. Following integration of comments during the pilot, a post-pilot version was confirmed by consensus, which contained 29 multiple choice questions split across 6 categories in total. Broadly, these categories can be summarised as 'Respondent Details', 'NGS Workflow', 'Variant Classification', 'Phenotypic Context', 'LIMs Status' and 'Central Submission'. These questions were added to an electronic survey format and user-tested for functionality by the CanVIG-UK leadership team prior to questionnaire release.

Participant Identification

Participants were identified from a list of persons previously involved with cancer susceptibility gene data submissions to Public Health England (PHE) or NHS Digital (NHSD), which was shared from the PHE data team with the CanVIG-UK leadership team. These persons were contacted to confirm that they were previously involved in one or more of the below activities:

- Variant Data Restructuring and Submission
- Design of Laboratory Information Management Systems (LIMs) or data flow between NGS outputs and LIMs
- Design of database structure for capture and storage of variant classifications

Involvement in these activities was deemed baseline for sufficient knowledge in completing the survey. The CanVIG-UK leadership also contacted the full CanVIG-UK membership to identify further individuals meeting these criteria, and in total 51 individuals were contacted to complete the survey. Individuals at the same laboratory were asked to co-ordinate and submit a single submission from their lab rather than submit individually in order to generate an overall answer to represent their lab workflow and prevent duplicate responses.

Web Survey (Pilot Phase 2)

The survey link was sent out to the selected pool of 51 individuals using SurveyMonkey (http://www.surveymonkey.com) and was live between February 2022 and April 2022. In the invitation email, participants were provided with details of the purpose behind the survey, how all their responses would be utilised as part of feedback to NHS England (with confirmation that individual participant or laboratory identifiers would not be included in any presentation of the data, including through any additional comments made). Incentives were not offered for participation. The investigators for the survey were confirmed as Prof Clare Turnbull, the CanVIG-UK team, and the PHE team, and participants were provided an approximate length of time the survey would take (around 10 minutes). Personal details (name, role, email address) were collected for the purposes of re-contacting respondents in order to deliver feedback in the first instance, to identify unique visitors or duplicate submissions, and to clarify responses given. This information was downloaded from SurveyMonkey and held for the duration of study analysis on a secure drive in a folder with access provided only to the investigators.

Questions were not randomised, and were split across 7 pages, one for each category plus an additional page for centralised data submissions. Number of questions per page varied as a result, between 1 and 11. All questions had a comment box and an 'Other' option. Most questions were required, however one question was reliant on specific selection from the

previous answer and was therefore optional to answer. All questions provided an 'other' or 'unsure' option. Respondents were able to save their progress and return to the survey to change answers or complete the survey at any point before the survey closed. If the same individual submitted multiple times, the most recent responses were retrieved and all other responses removed. Completion was reviewed after the questionnaires were submitted.

The web survey received a total of 27 responses, 18 of which were complete and from unique participants (17 responses from across all 16 submitting labs in England, and 1 response from the All Wales Medical Genomics Service). Following survey closure, a feedback meeting was held in May 2022 to discuss findings from this version of the survey and gather detailed feedback, which involved CanVIG-UK leadership, those involved with informatics at several Genomic Laboratory Hubs, and the survey respondents. This meeting highlighted that updates to several laboratory data management systems were ongoing, as well as responses which did not fit the multiple choices provided.

Email Survey (Final responses)
The survey was updated following the Web Survey to incorporate some common answers provided as 'Other' (for example, the addition of 'Patient Initials' and 'Patient Sex' as workflow identifiers in Question 1). One additional question was also included relating to NHS centralised data submission of variants of uncertain significance, bringing the total questions asked to 30.

To allow time for implementation of management system updates, the final iteration of the survey was sent out one year after the web survey, and was open between April and June 2023. To assist those responding, the final survey was sent to all 18 previous respondents as a pre-filled Microsoft Word document containing their previous answers. Any new questions or options were highlighted. Respondents were asked to review their answers in the context of new LIMs updates, and update or add to any comments made from the previous year. The invitation to complete this survey, in addition to the consent provided for the Web Survey, also described the investigators intent to publish responses from this final survey iteration.

By June 2023, 17 of the previous respondents had replied with completed questionnaires and had updated their answers as appropriate (94.4% response rate). 1 respondent did not reply as a member of their laboratory had already provided responses for the email survey. Responses were received as Microsoft Word documents, which were manually collated into a 'master' Microsoft Excel database for review.
