# Supplementary material for: Recommendations for laboratory workflow that better support centralised amalgamation of genomic variant data: findings from CanVIG-UK national molecular laboratory survey
Source: J Med Genet. 2023 Dec 22;61(4):305–12. doi: 10.1136/jmg-2023-109645 (PMC10982625; doi:10.1136/jmg-2023-109645)
Supplement: Supplementary data [file jmg-2023-109645supp001.pdf]

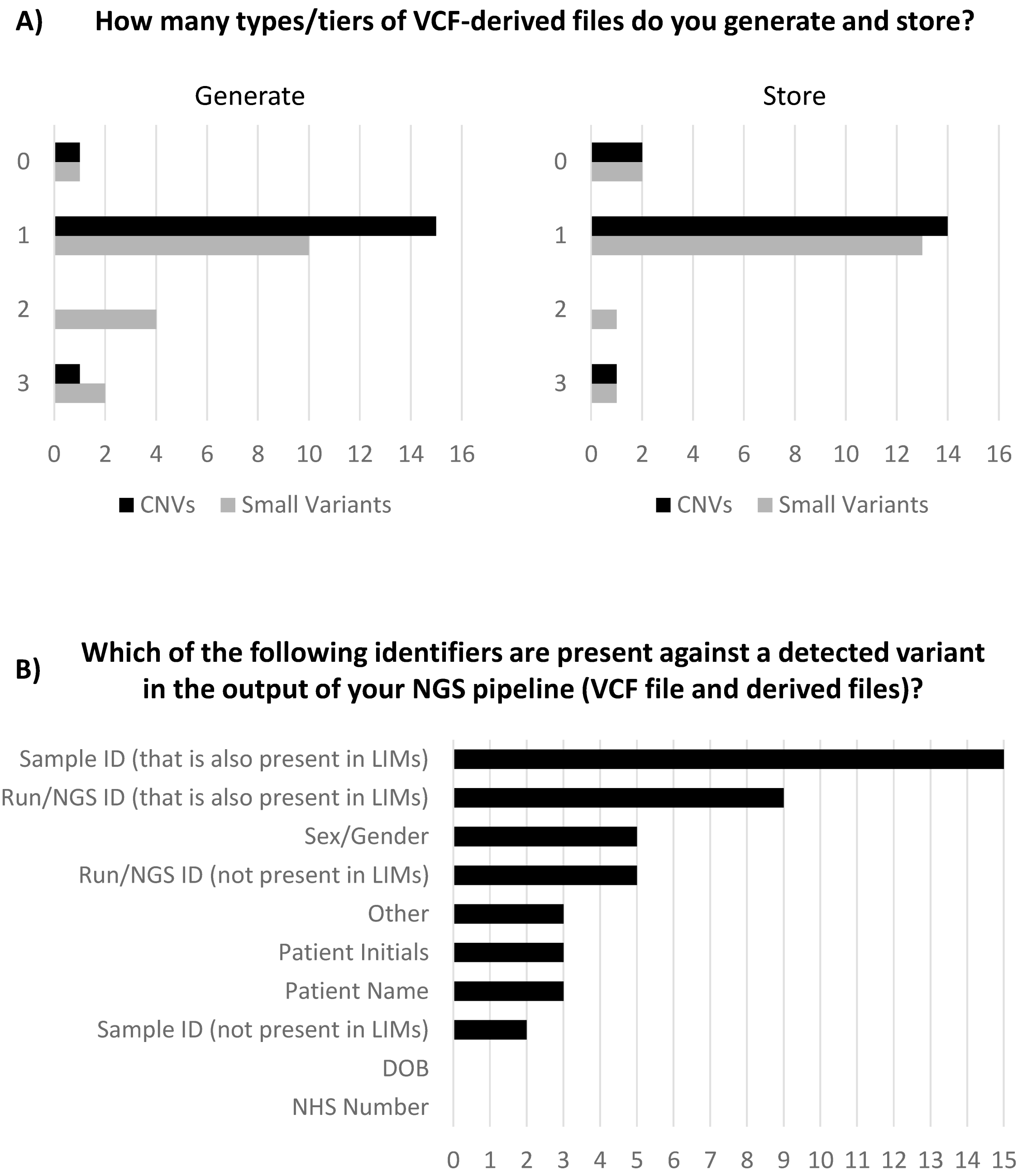

Supplementary Figure 1: Additional questions pertaining to variant workflows. A: Approaches to storing intermediary files generated as part of the workflow. B: The range of patient identifiers available within the workflow upstream of entry in the LIMs.

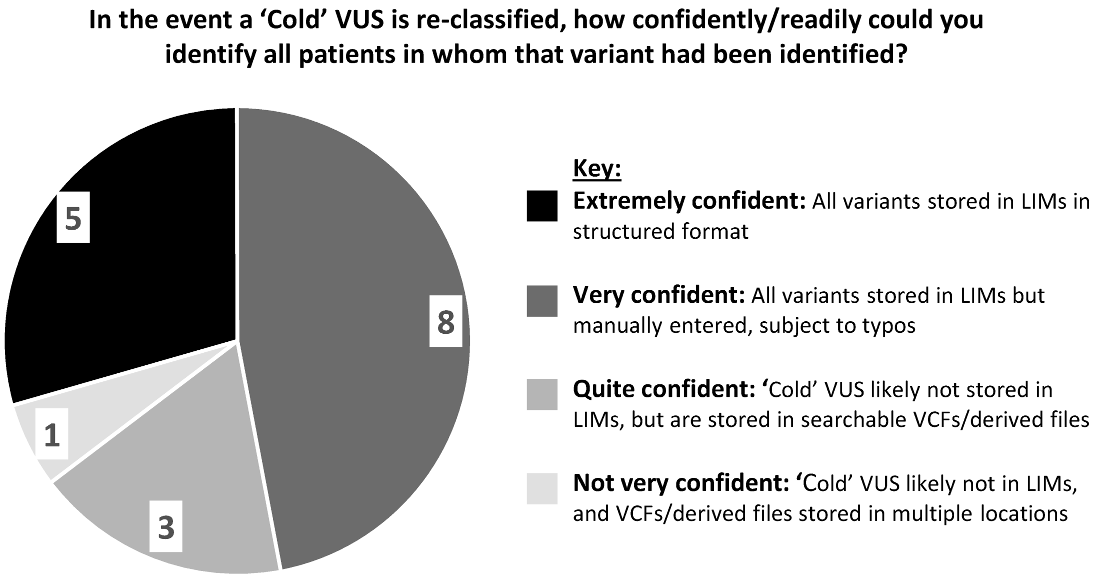

Supplementary Figure 2: Confidence of responding laboratories in ability to reliably retrieve 'cold' VUS variant information in the event of reclassification.
